# Clinical Analysis of the Frosch Approach in the Treatment of Posterolateral Tibial Plateau Fractures Combined with Lateral Tibial Plateau Fractures

**DOI:** 10.1111/os.13890

**Published:** 2023-09-14

**Authors:** Tangbo Yuan, Dawei Cai, Fei Yang, Zeyong Wang, Jian Qin

**Affiliations:** ^1^ Department of Orthopaedics, Sir Run Run Hospital Nanjing Medical University Nanjing China

**Keywords:** Frosch approach, Posterolateral, Surgical treatment, Tibial plateau fracture

## Abstract

**Objective:**

The treatment of posterolateral tibial plateau fractures is difficult, and providing sufficient exposure and effective fixation is a challenge. There is great controversy regarding the surgical approach for posterolateral tibial plateau fractures. The purpose of the study was to investigate the clinical effects of open reduction and internal fixation using the Frosch approach for the treatment of posterolateral tibial plateau fractures combined with lateral tibial plateau fractures.

**Methods:**

Data from 19 patients with posterolateral tibial plateau fractures combined with lateral tibial plateau fractures treated from May 2018 to January 2022 were retrospectively analyzed. There were nine men and 10 women, ranging in age from 22 to 62 years, with an average age of 45.6 years. All patients were treated using the Frosch approach. Under direct vision, the posterolateral and lateral fractures were reduced, and full bone grafting was performed. We reshaped the oblique “T” shaped plate for the distal radius and placed it on the posterolateral tibial plateau to fix the posterolateral fractures. The lateral inverted “L” shaped locking plate was placed on the lateral tibial plateau to fix the lateral tibial plateau fractures. Within 2 weeks after the operation, the patients were instructed to perform knee joint function exercises within 90°. At the last follow‐up, the Rasmussen radiological criteria were used to evaluate the effectiveness of fracture reduction and fixation. And the knee joint function was evaluated using Rasmussen functional score.

**Results:**

The operation time ranged from 100 to 180 min, with an average of 134.2 min; intraoperative blood loss ranged from 20 to 150 mL, with an average of 66.8 mL. The follow‐up duration ranged from 14 to 58 months, with an average of 36.2 months. There were no complications, such as vascular or nerve injury or incision infection. Fracture healing was achieved in all patients, and the healing time ranged from 10 to 14 weeks, with an average of 11.2 weeks. During the follow‐up period, there was no loosening or breakage of the internal fixation, varus or valgus deformity of the knee joint, re‐collapse of the articular surface, or instability of the knee joint. At the last follow‐up, the effectiveness of fracture reduction and fixation was excellent in 13 patients and good in six patients. And the knee joint function was excellent in 17 patients and good in two patients.

**Conclusion:**

The Frosch approach for open reduction and internal fixation in the treatment of posterolateral tibial plateau fractures combined with lateral tibial plateau fractures has a definite clinical benefit and is worthy of promotion and application.

## Graphical abstract

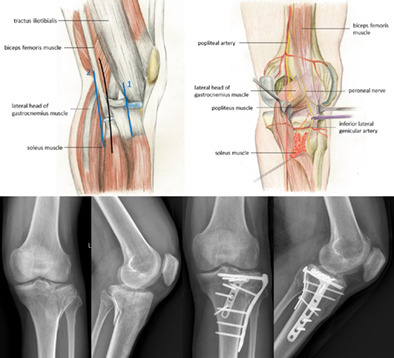

## Introduction

Tibial plateau fractures, also known as tibial condylar fractures, account for 1% to 2% of total fractures.^1^ Tibial plateau fractures involving the posterolateral condyle account for 15% of all tibial plateau fractures,^2^, ^3^ which are mostly caused by low‐energy injuries, such as sprains and falls.

Currently, the Schatzker classification is the most widely used classification method for tibial plateau fractures. However, this classification has limitations, such as limited guidance for the treatment of posterolateral fractures. Luo *et al*.^4^ proposed a three‐column classification theory of tibial plateau fractures based on CT scanning. This classification system can provide important guidance for the selection of surgical approaches and internal fixators for posterolateral fractures, and its reliability is higher than that of the Schatzker classification.^5^

A posterolateral fracture of the tibial plateau is a special intra‐articular fracture often associated with ligament and meniscal injuries.^6^ The most common postoperative complication of tibial plateau fractures is post‐traumatic osteoarthritis,^7^ which also decreases with improvements in surgical techniques. For tibial plateau fractures, restoring the flatness of the joint surface and alignment of the knee joint during surgery are key to preventing post‐traumatic osteoarthritis.^8^ Most tibial plateau fractures can be treated using standard reduction and fixation techniques; however, when posterolateral fractures are involved, owing to the obstruction of the posterolateral fibular head and the presence of important blood vessels, nerves, and other structures on the posterolateral side of the knee joint, many difficulties remain in reducing and fixing the posterolateral tibial plateau fracture fragments, making their treatment relatively challenging. There is great controversy regarding the surgical approach for posterolateral tibial plateau fractures, anterolateral approach, modified posterolateral approach, and posteromedial approach can all be used to treat posterolateral tibial plateau fractures.^9^, ^10^ The above approaches all have certain drawbacks and limitations, for instance, using traditional anterolateral approaches, it is difficult to reduce and fix posterolateral fractures under direct visualization. Many scholars have attempted to solve the problem of insufficient exposure of posterolateral tibial plateau fractures through the anterolateral approach. Therefore, finding a suitable surgical approach and providing sufficient reduction and effective fixation of the collapsed joint surface are key to preventing complications such as post‐traumatic osteoarthritis.

The Frosch approach can expose the posterolateral and lateral fracture fragments simultaneously through lateral single incision and double approaches. Through this approach, fracture fragments, especially posterolateral fracture fragments, can be effectively exposed, reduced, and fixed.

For the collapse of the posterolateral joint of the tibial plateau, especially posterolateral wall fracture and displacement, the fracture should be reduced and fixed with a posterolateral buttress plate. However, considering the complex anatomical of the posterolateral of the knee joint, the exposure range is limited, and there is a risk of injury to blood vessels and nerves.^11^ Many surgical approaches do not accommodate placement of the posterolateral buttress plate,^12^, ^13^, ^14^ which is the difficulty of clinical treatment at present.

The purpose of our study was as follows: (i) to explore the clinical effects of open reduction and internal fixation through the Frosch approach in the treatment of posterolateral tibial plateau fractures combined with lateral tibial plateau fractures; (ii) summarize the surgical techniques and precautions for this surgical approach; and (iii) provide a reference for the surgical approach and selection of internal fixators for such fractures.

## Materials and Methods

### Inclusion and Exclusion Criteria

Case inclusion criteria were: (i) patients with fresh posterolateral tibial plateau fractures combined with lateral tibial plateau fractures (within 3 weeks after fracture) who could cooperate with follow‐up and functional exercises; (ii) the knee joint function of the patient was normal before injury; (iii) patients with joint collapse and separation >3 mm; and (iv) complete follow‐up data of at least 1 year or more. Exclusion criteria: (i) patients with high anesthesia risk or low prognosis requirements and surgical contraindications; (ii) patients with open tibial plateau fractures; (iii) patients with vascular nerve bundle of the popliteal fossa (include popliteal artery, vein, and tibial nerve) injuries or osteofascial compartment syndrome; and (iv) complications affecting efficacy evaluation, such as hemiplegia, and psychoneurosis. This study was approved by the Ethics Committee of our hospital (2021‐SR‐038). All patients provided written informed consent.

### Preoperative Preparation

After admission, routine radiography, CT with 3D reconstruction, and MRI were performed to further clarify the combined injury. We elevated and immobilized the affected limb, applied local cold compression, measured the pulse in the dorsal foot artery of the affected limb, observed the blood supply to the affected limb, and prevented thrombosis with low‐molecular‐weight heparin sodium. Patients with tinea pedis were instructed to soak their feet in iodophor water preoperatively. The swelling of the affected limb subsided significantly, the skin wrinkled, and an internal fixation operation was performed after controlling for medical conditions and excluding patients based on surgical contraindications. Injected antibiotics (cefazolin sodium) were administered prophylactically 30 min before the operation. Before the operation, the lateral collateral ligament, fibular capitulum, and common peroneal nerve were located on the body surface, and the surgical incision was marked. Before surgery, patients with medical conditions were referred to the internal medicine and anesthesia departments for assistance in diagnosis and treatment.

### Surgical Technique

**FIGURE 1:**
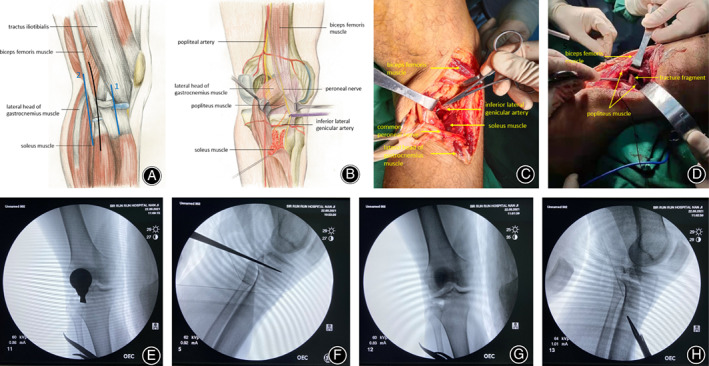
(A) Schematic diagram of the surgical incision and approach. Black line: skin incision. Blue line 1: anterolateral standard approach. Blue line 2: posterolateral approach between the soleus muscle and lateral head of the gastrocnemius muscle. (B) An illustration of the posterolateral corner of the tibial plateau. (C) The lateral inferior genicular artery should be exposed and ligated during the operation. (D) During the operation, the popliteal tendon needs to be cut to fully expose the posterolateral corner of the tibial plateau, and both ends of the popliteal tendon are marked with sutures to facilitate suturing at the end of the operation. (E, F) A wide osteotome is used to reduce the collapsed articular surface. (G, H) Intraoperative fluoroscopy shows that the collapsed articular surface has reached anatomical reduction. (A, B) is modified from Frosch *et al*.

**FIGURE 2:**
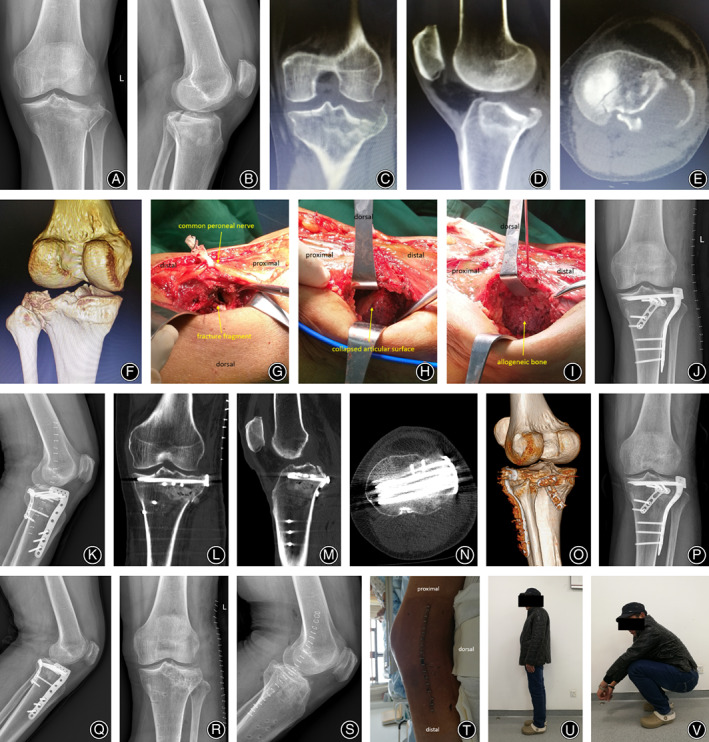
Typical case 1. A 58‐year‐old man was injured from a beating. (A–F) Preoperative X‐ray and CT images show a posterolateral tibial plateau fracture combined with a lateral tibial plateau fracture. (G–I) During the operation, the articular surfaces of the posterolateral tibial plateau and the lateral tibial plateau are obviously collapsed. After anatomical reduction of the articular surface, allograft bone is implanted in the bone defect area. (J–O) Postoperative X‐ray and CT images show an anatomical reduction of the tibial plateau fractures. (P, Q) Ten weeks after the operation, anteroposterior and lateral radiographs of the knee show that the fracture is completely healed. (R, S) One year after the operation, the internal fixation was removed. (T) The appearance of the patient's surgical incision. (U, V) The function of the patient's knee joint at the last follow‐up. (In this case, the posterolateral tibial plateau fractures were mainly the collapse of the posterolateral articular surface).

Combined spinal epidural anesthesia or vein‐inhalation mixed general anesthesia was administered. After the anesthesia had taken effect, the anterior and posterior drawer test and Bohler sign were performed on the affected knee joint, combined with a preoperative examination to clarify the diagnosis. The patient was placed in a lateral recumbent position, and the surgeon made a longitudinal straight incision about 15 cm in length along the axis of the fibula (Figure 1A), cut the skin, subcutaneous tissue, and deep fascia in turn, dissected and exposed the common peroneal nerve along the back of the long head of the biceps femoris and protected it (Figure 2G), separated and exposed the inferior lateral genicular artery and ligated it (Figure 1B,C), pulled the lateral head of the gastrocnemius muscle to the inside, exposed the fracture site, separated and exposed the popliteal tendon, and cut it. After cutting the posterolateral joint capsule and fixing it with a suture, the surgeon pulled upward to fully expose the posterolateral joint surface of the knee joint (Figure 1B,D). The periosteal stripping ion was used to strip 4 cm along the posterior edge of the tibial plateau to the distal end to avoid injuring the perforating branch of the popliteal artery (Figure 1B). The surgical incision was then pulled forward, the iliotibial bundle was cut along the direction of the muscle fiber and sharply separated to expose the lateral tibial plateau (Figure 1A) and separated backward to the fibular head. The joint capsule was cut under the lateral meniscus, and the articular surface was exposed. If the lateral tibial plateau fractures were split or with collapsed fractures, the split platform was partially turned over, and an osteotome was used to elevate the collapsed articular surface for reduction. If there were fractures in the posterolateral wall of the tibial plateau, it was also possible to use an osteotome to elevate the collapsed articular surface through the ruptured posterolateral wall (Figure 1E–H). If the lateral tibial plateau fractures were simple collapse fractures, a window was opened under the lateral tibial plateau to reduce the collapsed articular surface. Simultaneously, we observed a reduction in collapse of the posterolateral tibial plateau through the posterolateral approach. If necessary, the lateral and posterolateral fractures can be “interactively” reduced at the same time.

After confirming satisfactory reduction of the lateral and posterolateral tibial plateau under direct vision and fluoroscopy, allograft bone was implanted into the bone defect under the raised articular surface (Figure 2I). The lateral platform was fixed with an inverted “L” shaped locking plate (4.2 × 12.5 × 102 mm, with six holes in the trunk of the plate, Synthes, Solothurn, Switzerland) of the tibial platform, and the posterolateral platform was fixed with an oblique “T” shaped plate (2 × 10 × 62 mm, with four holes in the trunk of the plate, Synthes) to fix the distal radius fracture. C‐arm fluoroscopy confirmed that the collapse and widening of the platform were corrected, fracture reduction was satisfactory, and the position and length of the plate and screw were satisfactory. After verifying good stability of the knee joint, the incision was sutured layer by layer. Attention was directed to suturing and repairing the joint capsule and popliteal tendon, and the elastic bandage was appropriately pressurized. All surgeries were performed by the same surgeon.

### Postoperative Treatment

Intravenous prophylactic antibiotics (cefazolin sodium) were administered for 24–48 h after the operation, and X‐rays and CT scans of the knee joint were reexamined. The elastic bandages were pressed and wrapped until the surgical incision was dry, without exudation. Low‐molecular‐weight heparin was injected subcutaneously on the day after the operation to prevent deep venous thrombosis. Rivaroxaban was administered orally for 14 days after discharge. On the first day after the operation, the patients were instructed to perform isometric contraction and other functional exercises of the quadriceps femoris, and within 2 weeks after the operation, the patients were instructed to perform knee flexion and extension exercises within 90°. Six weeks after the operation, knee flexion function exercise was gradually increased, and the knee extension function was monitored. Before clinical healing of the fracture, the affected limb was maintained in a non‐weight‐bearing status. After clinical healing of the fracture, the affected limb was gradually allowed to bear weight until it was completely loaded.

### Follow‐up and Efficacy Evaluation

After 1, 2, 3, 6, and 12 months and every 6 months thereafter, regular outpatient reexamination was performed. During follow‐up, symptom improvement was evaluated, and physical examination was performed. Radiographs of the knee joint were routinely re‐examined (CT scans were performed when necessary) combined with physical examination to evaluate fracture healing and guide the patients in performing functional exercise.

Complications that occurred during the follow‐up period were also recorded, including vascular and nerve injury, incision infection, loosening or breakage of the internal fixation, varus or valgus deformity of the knee joint, re‐collapse of the articular surface, or instability of the knee joint, etc. At the last follow‐up, the effectiveness of fracture reduction and fixation of the patient's knee joint was evaluated using the Rasmussen radiological criteria, including platform collapse (6 points), platform widening (6 points), and knee varus (6 points), with a total score of 18 points: excellent (18 points), good (12–17 points), fair (6–11 points), and poor (<6 points).^15^ The Rasmussen functional score was used to evaluate knee joint function, including patient self‐evaluation and objective clinical examinations. The score comprises pain (6 points), walking ability (6 points), lack of knee extension (6 points), knee joint mobility (6 points), and knee joint stability (6 points), with a total score of 30 points; ≥27 points was considered excellent, 20–26 points was considered good, and 10–19 points was considered fair, and 6–9 points was considered poor.^15^

## Results

### General Results

According to the inclusion and exclusion criteria, 19 patients with complete follow‐up data were obtained, including nine men and 10 women, whose ages ranged from 22 to 62 years, with an average of 45.6 years. Surgery was performed in 10 patients on the left side and in nine patients on the right side. The causes of injury were falls in 12 patients, traffic injuries in four, falling injuries in two, and other injuries in one. According to the Schatzker classification, all 19 patients were classified as type II, and according to Luo *et al*.^4^ three column classification, all patients were classified as posterolateral column combined with lateral column fractures. Eight patients had fibular head or neck fractures, including three patients with common peroneal nerve injury. Seven patients had a meniscal injury, seven had a cruciate ligament injury, eight had a collateral ligament injury, and one had a tibiofibular syndesmosis injury (Table 1).

**TABLE 1:** Demographic characteristics of 19 patients with posterolateral tibial plateau fractures combined with lateral tibial plateau fractures.

Case number	Sex	Age (years)	Cause of injury	Affected limb	Preoperative time (days)	FH or FN fracture	CPN injury
1	Male	58	B	Left	2	No	No
2	Female	46	FFH	Left	5	Yes	No
3	Male	53	FFEV	Left	3	No	No
4	Male	55	FFEV	Left	12	No	No
5	Female	51	FFEV	Left	3	No	No
6	Male	22	TA	Left	2	No	No
7	Female	50	F	Left	2	Yes	No
8	Male	54	FFH	Left	5	No	No
9	Female	48	TA	Right	7	No	No
10	Female	50	FFEV	Right	3	Yes	No
11	Male	49	F	Left	3	Yes	No
12	Female	33	TA	Right	2	Yes	Yes
13	Male	33	FFEV	Left	4	Yes	No
14	Female	53	TA	Left	2	No	No
15	Female	41	FFEV	Left	9	Yes	Yes
16	Female	32	FFEV	Left	3	No	No
17	Male	39	F	Right	2	No	No
18	Male	62	F	Left	4	Yes	Yes
19	Female	38	FFEV	Left	5	No	No

Abbreviations: B, beaten; CPN, common peroneal nerve; F, falls; FFEV, falls from an electric vehicle; FFH, falls from a height; FH, Fibular head; FN, Fibular neck; TA, traffic accident.

The duration from injury to surgery was 2–12 days (average, 4.1 days). The operative time was 100–180 min (average, 134.2 min); and the intraoperative blood loss was 20–150 mL (average, 66.8 mL). The follow‐up duration ranged from 14 to 58 months, with an average of 36.2 months. Fracture healing was achieved in all patients, and the healing time was 10–14 weeks (average, 11.2 weeks) (Table 2).

**TABLE 2:** Clinical data of 19 patients with posterolateral tibial plateau fractures combined with lateral tibial plateau fractures.

Case number	Operation time (min)	Intraoperative blood loss (mL)	Follow‐up time (months)	Fracture healing time (weeks)	Knee extension/flexion (°)	Rasmussen radiological score	Rasmussen functional score
1	180	150	58	10	0‐0‐135	18	30
2	160	100	51	10	0‐0‐135	18	30
3	140	50	54	12	0‐0‐125	18	28
4	150	100	51	13	0‐0‐120	16	27
5	120	50	50	12	5‐0‐125	18	26
6	150	120	46	10	0‐0‐130	18	29
7	140	90	46	12	0‐0‐120	16	27
8	130	60	39	10	0‐0‐135	18	30
9	120	50	39	11	0‐0‐125	18	28
10	130	30	38	12	0‐0‐120	16	27
11	130	20	33	11	0‐0‐125	18	28
12	110	60	29	10	0‐0‐120	16	27
13	130	100	27	10	0‐0‐130	18	29
14	140	50	26	13	0‐0‐120	16	27
15	130	80	26	11	0‐0‐125	18	28
16	120	30	26	10	0‐0‐130	18	29
17	140	60	18	10	0‐0‐135	18	30
18	130	50	16	14	5‐0‐100	14	24
19	100	20	14	11	0‐0‐125	18	28

### Treatment of Combined Injuries

Eight patients with combined fibular head or neck fractures healed, while three patients with combined common peroneal nerve injury recovered after intraoperative release and symptomatic treatment with postoperative nutrition. Seven patients with meniscal injury underwent conservative treatment, seven patients with cruciate ligament injury had negative intraoperative drawer tests, eight patients with collateral ligament injury had negative intraoperative lateral stress tests, and one patient with upper tibiofibular joint injury underwent Kirschner wire elastic fixation. The internal fixation was removed one and a half years after surgery without any further dislocation.

### Radiological and Knee Joint Function Evaluation

At the last follow‐up, the Rasmussen radiological criteria were used to evaluate the effectiveness of fracture reduction and fixation: excellent in 13 patients and good in six patients. And the knee joint function was evaluated according to Rasmussen functional score: excellent in 17 patients and good in two patients (Table 2).

### Complications

There were no complications such as vascular and nerve injury, incision infection, skin necrosis and plate exposure during the perioperative period. And during the follow‐up period, there was no loosening or breakage of the internal fixation, varus or valgus deformity of the knee joint, re‐collapse of the articular surface, or instability of the knee joint.

**FIGURE 3:**
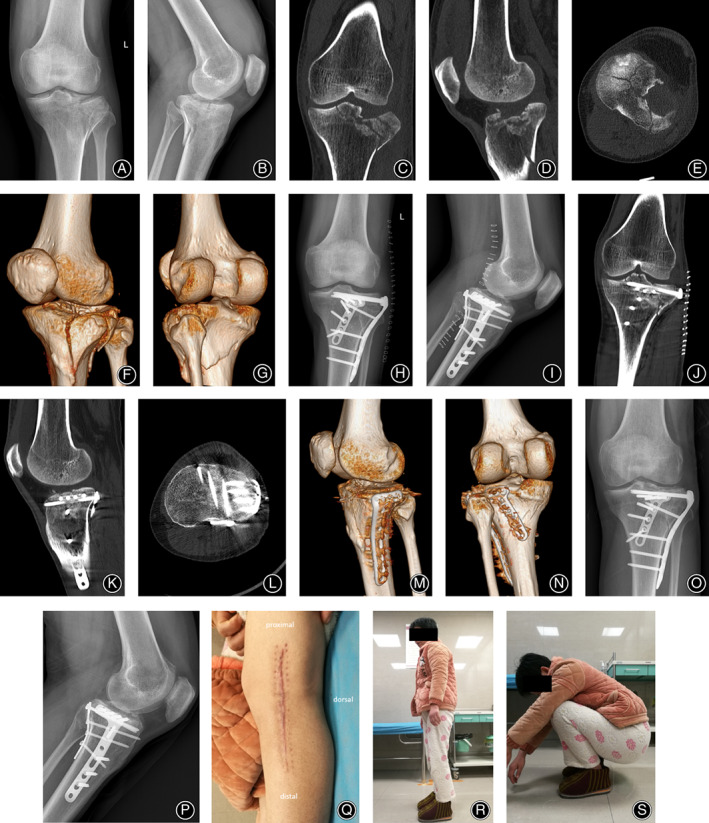
Typical case 2. A 46‐year‐old woman was injured by falling from a height. (A–G) Preoperative X‐ray and CT images show a posterolateral tibial plateau fracture combined with lateral tibial plateau fracture. The lateral tibial plateau has a split fracture with articular surface collapse, and the posterolateral tibial plateau has a posterolateral wall fracture with the fracture line extending to the posteromedial wall. (H–N) Postoperative X‐ray and CT images show an anatomical reduction of the tibial plateau fractures. (O, P) Eleven weeks after operation, the anteroposterior and lateral radiographs of the knee show that the fracture is completely healed. (Q) The appearance of the patient's surgical incision. (R, S) The function of the patient's knee joint at the last follow‐up. (In this case, the posterolateral tibial plateau fractures were mainly posterolateral wall fractures).

A 53‐year‐old female patient showed evidence of ~2 mm steps on the articular surface of the posterolateral platform on a postoperative CT scan. She did not have obvious pain even when squatting. No obvious signs of post‐traumatic osteoarthritis were found on follow‐up after 26 months. A 62‐year‐old male patient had good fracture reduction and healing but did not follow the doctor's instructions for timely outpatient review, did not participate in good functional exercise, and had a range of motion of the knee joint of 5°–100°. Typical cases are shown in Figures 2 and 3.

## Discussion

The 19 patients in our study were all treated surgically through the Frosch approach. These patients did not experience complications such as vascular or nerve injury during the surgery, and there were no postoperative complications such as incision infection, loosening or breakage of the internal fixation, re‐collapse of the articular surface, or instability of the knee joint. Among the 19 patients, 18 achieved anatomical reduction, and one patient showed evidence of ~2 mm steps on the articular surface of the posterolateral platform on a postoperative CT scan. All patients had satisfactory knee joint function recovery.

### Injury Mechanism and Diagnosis of Posterolateral Tibial Plateau Fractures

The typical injury mechanism of patients in our study is that the knee joint suffers from combined axial and valgus violence in the flexion or semi‐flexion positions.^16^ As the lateral femoral condyle is stronger than the posterolateral condyle of the tibial plateau, it usually causes a split or collapse fracture of the posterolateral tibial plateau on the coronal plane.^17^ Among the 19 patients in this study, eight were injured by riding an electric bicycle, and seven of them were injured in the left knee joint. The reason for the high proportion of left limb injuries is that people instinctively use the left lower limb for support when they fall while riding an electric bicycle.

A simple posterolateral fracture of the tibial plateau may appear hidden on radiography; sometimes only the local density decreases or increases, making diagnosis difficult.^18^, ^19^ When radiography reveals a fibular head and neck fracture, it is necessary to consider whether the patient has a posterolateral tibial plateau fracture.^20^ According to Biz *et al*., detailed preoperative radiological imaging and physical examination are crucial for tibial plateau fractures, especially for posterolateral tibial plateau fractures.^21^ In this study, all patients had undergone radiography, CT scan with 3D reconstruction, and MRI examination of the knee joint before surgery. After anesthesia, anterior and posterior drawer tests and the Bohler sign of the affected knee joint were performed to further clarify the diagnosis. Combined with intraoperative exploration, missed diagnoses and mistreatment can be avoided.

### Selection of Operative Approach for Posterolateral Tibial Plateau Fractures

In the past, posterolateral fractures of the tibial plateau were treated by opening a window at the proximal tibial metaphysis through the anterolateral or lateral approach, selecting an appropriate position to elevate and reduce the collapsed articular surface with a periosteal stripper, followed by bone grafting in the space below the tibial plateau, and finally fixation with a lateral plate.^22^ However, the use of lateral plates to fix posterolateral bone fragments has a major limitation in that the screws cannot be fixed to the posterior area of the tibial plateau. At present, an increasing number of surgeons choose to directly expose posterolateral bone fragments and perform reduction and fixation so that they can be reduced under direct vision.^11^, ^12^, ^23^, ^24^ After reduction, they can be fixed with posterolateral buttress plates. Particularly, for patients with posterolateral articular surface collapse and posterolateral wall fractures, it has definite advantages over the anterolateral approach. Common surgical approaches used to place the posterolateral buttress plate of the tibial plateau include posterolateral approach, Frosch approach, fibular head osteotomy approach, and posteromedial approach. The anatomical position of bone fragments on the articular surface of the posterolateral tibial plateau is deep, and the local anatomical structure is complex. The anterior tibial artery is relatively fixed at the position where it passes through the interosseous membrane hole, and the minimum distance from the articular surface of the lateral tibial plateau is only 27 mm.^25^

Orapiriyakul *et al*. found that compared to the posteromedial approach, the posterolateral approach has more advantages in treating posterolateral tibial plateau fractures. For the posteromedial approach, the blind area on the lateral platform that can only be exposed through the posterolateral approach starts at ~43.72% of the width of the tibial lateral platform and ends at 81.41%. When a fracture is in this area, it is recommended to use a posterolateral approach.^26^ Zhang *et al*. compared the visible area of the posterolateral tibial plateau exposed through different surgical approaches. The results showed that the average exposed area of the posterolateral tibial plateau through the Carlson posterolateral approach, Frosch approach, and fibular head osteotomy approach were (722.11 ± 18.79), (994.22 ± 26.92), and (1248.20 ± 18.08) mm^2^ respectively. Although the exposed area of the Frosch approach was less than that of the fibular head osteotomy approach, the Frosch approach did not require osteotomy, moreover, fractures of the lateral tibial plateau can be reduced and fixed through the same approach.^27^

### Advantages, Disadvantages, and Surgical Techniques of Frosch Approach

In 2010, Frosch *et al*. reported that AO, B3, C1, and C3 tibial plateau fractures can be treated using a modified lateral or posterolateral approach. The skin incision was made at the axis of the fibular head, and the deep part was entered from the anterior and posterior spaces of the fibular head, exposing the anterior and posterior sides of the tibial plateau, respectively. The free common peroneal nerve can simultaneously expose fractures of the lateral and posterolateral condyles. They treated a total of seven patients; six patients were followed up, and the medium‐term effect was good.^28^ The Frosch approach is very suitable for cases of posterolateral tibial plateau fractures combined with lateral tibial plateau fractures. For patients with multiple fractures, such as those with floating knee injuries,^29^ if posterolateral tibial plateau fractures are combined with lateral tibial plateau fractures, we believe that the Frosch approach can also be used to treat such fractures. However, when the Frosch approach is used to treat tibial plateau fractures, patients must remain in a lateral recumbent position. If fractures of the medial tibial plateau or other areas are also present, the patients' position must be changed during surgery.

The advantages of the Frosch approach in the treatment of posterolateral tibial plateau fractures are as follows: (i) this approach can be used to simultaneously reduce and fix the lateral and posterolateral tibial plateau fractures through the anterior and posterior space, taking into account the exposure needs of the anterolateral and posterolateral incisions, without making two incisions, which not only reduces surgical trauma but also reduces the risk of skin flap necrosis and plate exposure between the two adjacent incisions; (ii) combined common peroneal nerve injuries can be treated simultaneously; (iii) compared with other surgical approaches that expose posterolateral fractures of the tibial plateau, the Frosch approach has a larger exposure range for posterolateral fractures of the tibial plateau, which is more conducive to the reduction of posterolateral fractures and the placement of posterolateral buttress plates and does not require fibular head osteotomy, thus avoiding the possibility of nonunion and re‐displacement of fractures caused by fibular head osteotomy; and (iv) the incision is located on the outside of the knee joint, avoiding scarring of the incision, which affects knee joint flexion and extension. The Frosch approach also has disadvantages: (i) the posterolateral anatomical structure of the tibial plateau is complex; the operation is difficult, and the requirements for the surgeon are high. The surgeon should be familiar with the anatomical structure of the knee joint and have extensive experience in knee joint surgery; and (ii) it is difficult to judge the affected lower limb alignment and perform intraoperative fluoroscopy when the patient is in the lateral position.

The operational skills and precautions for the Frosch approach are as follows: (i) treatment of the lateral inferior genicular artery—during the operation, the lateral inferior genicular artery should be exposed and ligated; (ii) treatment of the popliteal tendon—the popliteal tendon should be cut during the operation, and a 1‐0 absorbable suture should be used to mark it in advance before cutting to facilitate the reconstruction of the popliteal tendon after the completion of fracture reduction and fixation; (iii) exposure of the posterolateral articular surface of the tibial plateau and fracture fragments—the posterolateral articular capsule was partially opened, and reduction of the posterolateral articular surface of the tibial plateau in the space below the meniscus was observed; (iv) Fracture reduction skills—the lateral and posterolateral fracture fragments can be exposed simultaneously during the operation, and anterior–posterior “interactive” reduction is adopted. If the articular surface collapses, the use of a thin bone knife is recommended to elevate and reduce the articular surface to avoid compressing the surrounding bone during prying reduction. After the bone fragments are well reduced, the bone defect area must be supported by an allograft bone graft; and (v) selection and placement techniques of plate—due to the presence of the anterior tibial artery emanating from the popliteal artery 4–5 cm below the articular surface of the tibial plateau, which increases the difficulty of exposing the distal of tibial and placing the posterolateral buttress plate, we chose an oblique “T” shaped plate for the distal radius on the same side.

After placing the proximal screw holes of the plate parallel to the articular line, the distal plate was naturally placed obliquely inward to avoid danger zones. Before placing the plate, it was necessary to shape the plate. Because of the deep exposure of the posterolateral side, sleeve protection was used when drilling holes to place screws to avoid damage to the blood vessels and nerves. For lateral tibial plateau fractures, we chose an anterolateral inverted “L” shaped plate of the tibial plateau. The proximal portion of the plate was placed as close to the joint line as possible so that the proximal row of screws was inserted as close to the subchondral bone as possible. This also reserved space for the implantation of the proximal row of screws in the posterolateral plate to avoid interference between the screws. In this way, the proximal row of screws of the lateral and posterolateral plates were inserted in a vertical direction, laying a “bamboo raft” supporting effect on the joint surface. The support strength of the joint surface was obviously improved, which is conducive to early functional exercise of patients after surgery.

The posterolateral tibial plateau has a unique local biomechanical environment.^30^ Posterolateral tibial plateau bone fragments that have not been reduced mainly affect knee joint stability in the flexion state. The posterolateral tibial plateau is not fully loaded during knee joint flexion. The posterolateral tibial plateau only bears the role of conducting stress when the knee joint is flexed >90°.^31^, ^32^ When the fixation strength of the posterolateral tibial plateau fracture is insufficient, patients must perform functional exercises within 90° of the knee joint under the protection of a knee joint hinge brace within 6 weeks after surgery so that the posterolateral tibial plateau does not bear the role of conducting stress within 6 weeks after surgery.^33^ In this study, the posterolateral tibial plateau was fixed with a buttress plate with a high fixation strength so that the patient could perform functional exercises of the knee joint above 90° during the early postoperative period. During the follow‐up period, there was no fracture re‐displacement in this group of patients. At the last follow‐up, the knee joint function was evaluated according to Rasmussen functional score: excellent in 17 cases (89.5%) and good in two cases. Fixation of posterolateral tibial plateau fractures with posterolateral buttress plates can provide effective stability. Immediate postoperative knee flexion and extension exercises exceeding 90° did not cause fracture displacement or joint surface collapse.

### Strengths and Limitations

The strength of this study is that all patients had posterolateral tibial plateau fractures combined with lateral fractures, avoiding the impact of fractures of other columns of the tibial plateau on clinical efficacy evaluation. The disadvantage of this study is that, as a retrospective study, the number of cases was small, and the follow‐up time was short. The extent to which posterolateral tibial plateau fractures have a significant impact on knee joint function is not clear. There was no control group. A more prospective comparative study on the injury mechanism and surgical treatment of posterolateral tibial plateau fractures will help clarify the effectiveness and incidence of complications of the surgical techniques used and verify their practicality and safety.

## Conclusion

In summary, the Frosch approach considers the exposure requirements of anterolateral and posterolateral incisions and can simultaneously handle fractures of the lateral and posterolateral tibial plateaus without fibular osteotomy, thus avoiding incision and soft tissue complications caused by dual anterolateral and posterolateral incisions of the tibial plateau. However, the Frosch approach is relatively difficult, and the operator should be familiar with the anatomical structure of the knee joint and have extensive experience in knee joint surgery.

## Author Contributions

Study design and supervision: Tangbo Yuan and Jian Qin. Acquisition of data: Dawei Cai, Fei Yang and Zeyong Wang. Analysis and interpretation of the data: Tangbo Yuan and Jian Qin. Patient follow‐up: Dawei Cai and Fei Yang. Image analysis: Tangbo Yuan and Zeyong Wang. Drafting of the manuscript: Tangbo Yuan and Fei Yang. All authors contributed to the review and revision of the manuscript. All authors read and approved the final manuscript.

## Funding Information

This study was supported by 2022 Nanjing Health Science and Technology Development Special Fund Project (No. YKK22207).

## Conflict of Interest Statement

The authors declare that there are no conflicts of interest.

## Ethics Statement

This study was granted approval by the ethics committee of Sir Run Run Hospital affiliated to Nanjing Medical University (No. 2021‐SR‐038). All patients provided written informed consent.
